# Intra-dialytic hypertension is associated with high mortality in hemodialysis patients

**DOI:** 10.1371/journal.pone.0181060

**Published:** 2017-07-25

**Authors:** Chi-Young Choi, Jae Seok Park, Kyu Tae Yoon, Hyo Wook Gil, Eun Young Lee, Sae Yong Hong

**Affiliations:** Department of Internal Medicine, Soonchunhyang University Cheonan Hospital, Cheonan, Korea; Hospital Universitario de la Princesa, SPAIN

## Abstract

**Background:**

Intra-dialytic hypertension (IDH) is emerging as an important issue in hemodialysis patients. Its risk factors and clinical outcomes are unclear.

**Methods:**

A total of 73 prevalent hemodialysis patients were enrolled. They included 14 (19.2%) patients with baseline IDH and 59 patients without IDH. Their clinical parameters, laboratory parameters, and mortality were investigated over 78 months.

**Results:**

The risks factor of IDH included low serum potassium levels, low ultrafiltration, and low arm muscle area. Lower median survival was evident in the IDH group compared to the non-IDH group, but was not significantly different. After adjusting for relevant confounders for age, the IDH group displayed 2.846 times higher mortality rate than the non-IDH Group (adjusted hazard ratio: 2.846; 95% confidence interval: 1.081–7.490; *P* = 0.034).

**Conclusion:**

IDH is associated with high mortality in hemodialysis patients. Clinicians should be aware of the risk factors. Future research studies are needed to explore the mechanisms involved in the association between IDH and mortality.

## Introduction

The prevalence of hypertension in patients undergoing hemodialysis ranges from 65% to 85% [1]. Hypertension results in cardiovascular complications in hemodialysis patients [2]. It is also a powerful predictor of death [3]. Intra-dialytic hypertension (IDH), a unique form of hypertension observed in hemodialysis patients, has recently emerged as an important issue.

In general, systolic blood pressure (SBP) decreases during hemodialysis. However, 8–15% of patients have IDH, which is an opposing trend to SBP [4,5]. Although the definition of IDH has not been firmly established, several defining criteria have been suggested, including increased mean arterial blood pressure exceeding 15 mmHg during or immediately after hemodialysis [6], SBP increase>10 mmHg from pre-dialysis to post-dialysis [7], development of hypertension during the second or third hour of hemodialysis after significant ultrafiltration [8], increased blood pressure that is resistant to ultrafiltration [9], and aggravation of pre-existing hypertension or development of *de novo* hypertension with the administration of erythropoietin-stimulating agents (ESAs) [10].

IDH has high mortality and morbidity rates [7]. However, the definite cause of IDH remains unclear. Proposed causes include sympathetic overactivity [4], volume overload [11], renin-angiotensin-aldosterone system activation [12], endothelial dysfunction [13], elimination of antihypertensive drugs by hemodialysis [14], ESAs [15], and dialytic sodium gradient [16]. However, none of these can clearly explain the etiology of IDH.

In the present study, we aimed to identify factors related to the development of IDH and ascertain the relationship between IDH and mortality in hemodialysis patients.

## Methods

### Patients

We screened 108 adult patients with end-stage renal disease (ESRD) in a single hemodialysis unit between June 2005 and September 2005. Thirty five patients were excluded because of incomplete data or loss to follow-up. A total of 73 patients were divided into the IDH group and non-IDH group. We used findings from a recently publication [13] to define IDH. The definition criteria were an increase in post-dialytic SBP >10 mmHg compared to pre-dialytic SBP in at least four of six consecutive hemodialysis sessions, and an absence of an intra-dialytic decline in SBP.

### Study design and data collection

This retrospective cross-sectional study was designed to determine clinical parameters, laboratory parameters, dialysis-related factors, and mortality. The study design was approved by the Institutional Review Board of Soonchunhyang University Cheonan Hospital, Cheonan, Korea (IRB approval number 2012–45). All patients provided their written informed consent before entering the study. The blood level of laboratory parameters was checked in 2005 and reviewed in 2016.

Clinical parameters included age, sex, height, duration of hemodialysis, underlying disease, left ventricular hypertrophy, dose of ESAs and antihypertensive drugs, body mass index (BMI), arm muscle area (AMA), and normalized protein catabolic rate (nPCR). Laboratory parameters obtained from pre-dialytic measurements included albumin, urea nitrogen, creatinine, hemoglobin, ferritin, sodium, potassium, bicarbonate, calcium, phosphorus, intact parathyroid hormone, and total cholesterol. Plasma renin activity, serum aldosterone, epinephrine, and norepinephrine values were checked before and after hemodialysis in all patients with IDH and 57 of 59 patients (97%) without IDH. Commercially available radioimmunoassay kits were used to measure plasma concentration of renin (Renin Riabead; Dainabot, Tokyo, Japan), aldosterone (Immunotech SA, Marseille, France), and epinephrine and norepinephrine (IBL, Hamburg, Germany). Dialysis-related factors included pre- and post -dialytic body weight, inter-dialytic weight gain, pre- and post-dialytic SBP, ultrafiltration volume, and urea [(clearance x time) / volume; Kt/V].

### Statistical analyses

Continuous data are expressed as mean ± standard deviation or mean ± standard error. Categorical data are expressed as frequencies (number of cases and percentages). Differences were determined with independent *t* test or Mann–Whitney *U* test for continuous data. Pearson’s chi-square test or Fisher’s exact test were used to compare categorical data.

Risk factors of IDH were identified with regression analysis. Survival curves were constructed using Kaplan-Meier estimates with comparisons between curves based on log-rank x2 statistic. IDH was related to all-cause mortality using univariable and multivariable Cox proportional hazard regression analyses in consideration of clinically plausible interactions. The primary end point was death. Time was the period from the beginning of the follow-up to the primary end point. The proportional hazard assumption was confirmed by inspecting log (−log [survival]) curves and examining time-dependent covariates. SPSS version 14.0 software (SPSS Inc., Chicago, IL, USA) was used for statistical analyses. In all statistical analyses, *P* < 0.05 was considered statistically significant.

## Results

### Baseline characteristics

**Fig 1:**
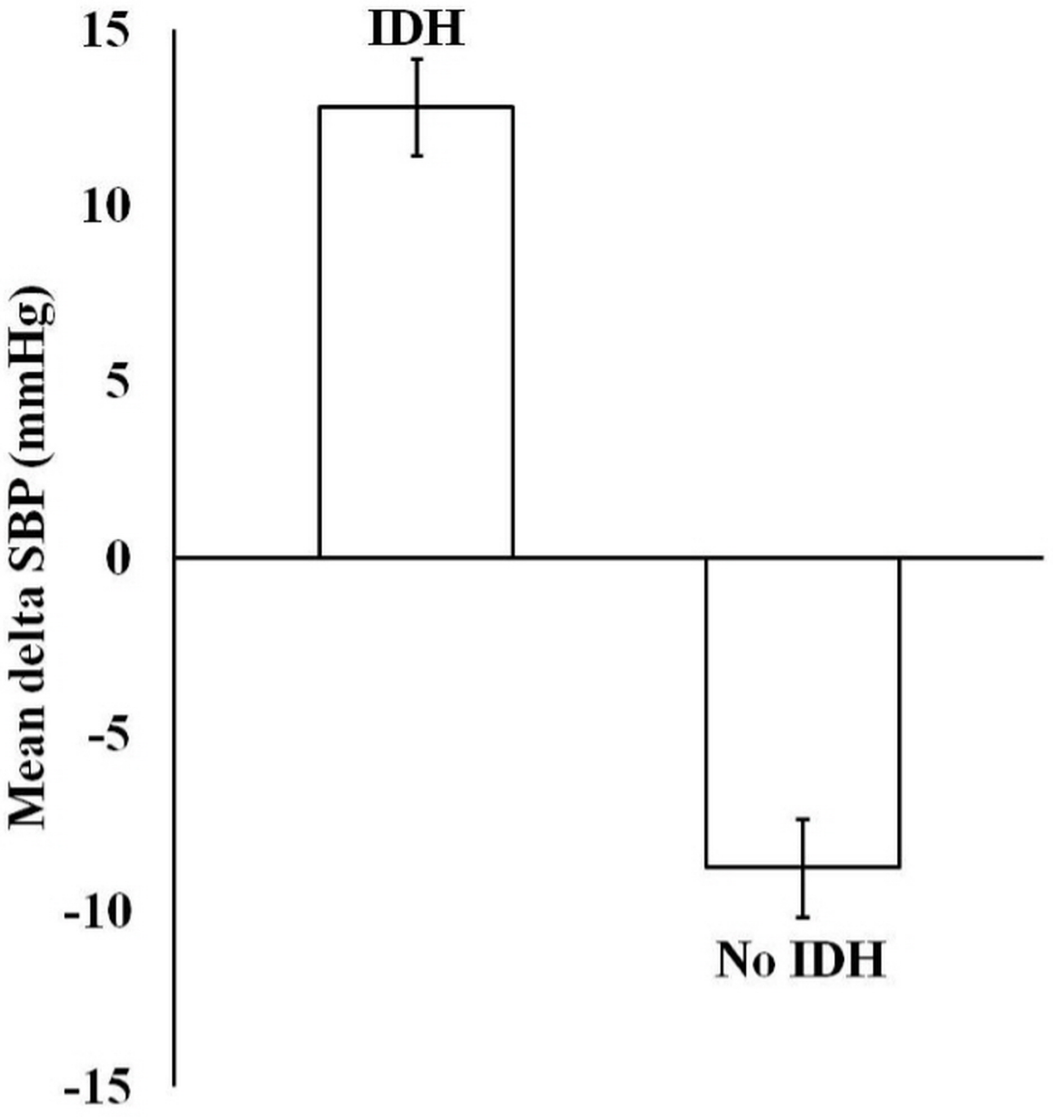
Mean delta SBP (post-dialytic SBP minus pre-dialytic SBP) of six consecutive hemodialysis sessions. The mean delta SBP in the IDH group was significantly higher than that in the non-IDH group (mean ± standard error, 12.78 ± 1.37 *vs* -9.42 ± 1.40; 95% confidence interval: 14.13 to 11.43 *vs*. -3.98 to -14.86; *P* < 0.001). SBP, systolic blood pressure; IDH, intradialytic hypertension.

IDH was prevalent in 14 (19.2%) of 73 patients. The mean delta SBP (post-SBP minus pre-dialytic SBP) value for six consecutive hemodialysis sessions in the IDH group was significantly higher than that in the non-IDH group (mean ± standard error, 12.78 ± 1.37 *vs*. -9.42 ± 1.40; 95% confidence interval [CI]: 14.13 to 11.43 *vs*. -3.98 to -14.86; *P* < 0.001) (Fig 1).

The mean age of the 73 patients was 54 years. Of the 73 patients, 41 (56.2%) were males. The causes of ESRD included diabetes mellitus (DM; *n* = 21), hypertension (*n* = 24), chronic glomerulonephritis (*n* = 18), polycystic kidney disease (*n* = 2), and unknown (*n* = 8). DM accounted for 28.8% of the causes of ESRD. The proportion of diabetic patients was relatively low because all unstable patients were excluded. Records at this center established that the prevalence of DM among the ESRD patients was 18% in 1992 and 49% in 2009. Since the number of diabetic patients has increased rapidly since 2000, the proportion of diabetic patients is likely to be lower in 2005 than in the present [17]. Of the 73 patients, 58 (79.5%) had hypertension, which was treated with regular antihypertensive medication. Thirty nine patients had left ventricular hypertrophy, including 7 patients in the IDH group and 32 patients in the non-IDH group. The mean pre- and post- dialytic body weights were 50.4 and 47.5 kg, respectively. The mean inter-dialytic weight gain was 2.8 kg. Mean ultrafiltration volume was 3.2 L. No significant differences in baseline characteristics were found between the two groups (Table 1).

**Table 1:** Baseline characteristics of patients.

Parameters	IDH (*n* = 14)	Non-IDH (*n* = 59)	*P*
**Age (years)**	54.1 ± 13.1	54.0 ± 13.0	0.982
**Male, *n* (%)**	11 (78.5%)	30 (50.8%)	0.077
**Target weight (kg)**	52.8 ± 8.0	57.9 ± 10.8	0.098
**Kt/V urea**	1.67 ± 0.65	1.68 ± 0.68	0.967
**SBP (mmHg)**			
Pre-dialytic	139.3 ± 14.9	140.0 ± 21.7	0.908
Post-dialytic	141.4 ± 15.1	131.7 ± 19.4	0.084
**DBP (mmHg)**			
Pre-dialytic	80.0 ± 7.8	79.5 ± 9.4	0.852
Post-dialytic	80.7 ± 8.3	78.6 ± 9.2	0.443
**Albumin (g/L)**	38 ± 5.0	39 ± 4.7	0.387
**Creatinine (umol/L)**	753.9 ± 225.2	844.5 ± 281.8	0.267
**Calcium (mmol/L)**	2.13 ± 0.19	2.15 ± 0.19	0.765
**Phosphorus (mmol/L)**	1.19 ± 0.36	1.37 ± 0.51	0.215
**Intact PTH (ng/L)**	90.8 ± 75.6	88.0 ± 101.5	0.925
**Hemoglobin (g/L)**	100 ± 7	103 ± 11	0.269
**Erythropoietin doses (U/week)**	6714 ± 4322	4361 ± 4558	0.084

Data are presented as mean ± standard deviation or number (percentage).

IDH, intra-dialytic hypertension; SBP, systolic blood pressure; DBP, diastolic blood pressure; PTH, parathyroid hormone.

### Clinical parameters, laboratory parameters, and dialysis-related factors

Serum potassium levels, inter-dialytic weight gain, ultrafiltration volume, urea nitrogen, BMI, AMA, and nPCR were significantly lower in the IDH group compared to those in the non-IDH group (Student’s t-test; Table 2). Multivariate logistic regression was performed on factors with P < 0.05. Serum potassium level, ultrafiltration volume, and AMA remained significant risk factors for IDH. The odds ratio (OR) of IDH was 0.089 (95% CI: 0.017–0.476, *P* = 0.005) for each increase of 1.0 mmol/L in serum potassium level. OR was 0.386 (95% CI: 0.166–0.894, *P* = 0.026) for each increase of 1.0 L in ultrafiltration volume and 0.887 (95% CI: 0.800–0.982, *P* = 0.021) for each increase of 1.0 cm^2^ in AMA (Table 3).

**Table 2:** Laboratory parameters and dialysis-related factors in the IDH and non-IDH groups.

Parameters	IDH	Non-IDH	*P*
**Sodium(mmol/L)**	138.21 ± 4.154	137.0 ± 16.6	0.463
**Potassium (mmol/L)**	4.7 ± 0.5	5.2 ± 0.7	0.003
**Bicarbonate (mmol/L)**	21.5 ± 4.2	22.4 ± 3.1	0.471
**Pre**-**dialytic body weight (kg)**	55.6 ± 8.9	61.5 ± 11.3	0.072
**Post**-**dialytic body weight (kg)**	53.3 ± 8.0	58.6 ± 11.0	0.098
**Inter**-**dialytic weight gain (kg)**	2.4 ± 1.2	3.0 ± 0.8	0.032
**Ultrafiltration (L)**	2.7 ± 1.1	3.5 ± 0.8	0.002
**Urea nitrogen (mmol/L)**	15.2 ± 6.6	19.4 ± 6.1	0.026
**BMI (kg/m**^**2**^**)**	19.6 ± 2.4	21.8 ± 3.2	0.017
**AMA (cm**^**2**^**)**	25.5 ± 6.0	32.2 ± 10.4	0.028
**nPCR (g**∙**kg**^**-1**^∙**day**^**-1**^**)**	0.69 ± 0.12	0.86 ± 0.24	0.011
**Hemoglobin(g/dL)**	9.98 ± 0.74	10.31 ± 1.05	0.269
**Hematocrit(%)**	30.3 ± 2.62	31.81 ± 3.19	0.126

Data are presented as mean ± standard deviation.

IDH, intra-dialytic hypertension; BMI, body mass index; AMA, arm muscle area, nPCR, normalized protein catabolic rate.

**Table 3:** Multivariate logistic regression analysis in the IDH groups (Odds ratio, 95% confidence intervals).

Parameters	P	Odds Ratios(95% confidence intervals)
**Potassium(mmol/L)**	0.0046	0.089(0.017–0.476)
**Inter-dialytic weight gain(kg)**	-	-
**Ultrafiltration**	0.0026	0.386(0.166–0.894)
**Urea nitrogen(mmol/L)**	-	-
**BMI (kg/m2)**	-	-
**AMA (cm2)**	0.021	0.887(0.800–0.982)
**HCO3**	0.09	1.127(0.979–1.298)
**nPCR (g∙kg-1∙day-1)**	-	-

BMI, body mass index; AMA, arm muscle area; nPCR, normalized protein catabolic rate.

### Renin-angiotensin-aldosterone system and sympathetic activity

Serum aldosterone levels before and after hemodialysis were significantly lower in the IDH group than those of the non-IDH group. However, there was no significant difference in plasma renin activity (pre- or post- dialytic), epinephrine level, or norepinephrine level between the two groups (Table 4). To understand the confounding effect of antihypertensive drugs, we classified these drugs into two categories: potassium-related drugs (angiotensin receptor blockers, angiotensin-converting enzyme inhibitors, and beta-blockers) and potassium-unrelated drugs (calcium channel blockers and vasodilators like minoxidil). Patients were then subdivided according to whether potassium-related drugs were prescribed. Diuretics were not used in any of these patients. In the IDH group, potassium-related drugs were prescribed to 12 patients, whereas 2 patients were not prescribed any antihypertensive drug. In the non-IDH group, potassium-related drugs were prescribed to 42 patients, whereas 12 patients received potassium-unrelated drugs and 5 patients did not receive any antihypertensive drug. There was no significant difference in the frequency with which potassium-related drugs were prescribed between the IDH group and the non-IDH group according to Fisher’s exact test. The difference in the rates of hypertensive dialyzable drugs (minoxidil and Iodixanol) and non-dialyzable drugs(valsartan, losartan, candesartan, telmisartan, carvediol, nifedipine, and amlodipine) between the IDH and non-IDH groups was not statistically significant.

**Table 4:** Renin–angiotensin–aldosterone system and sympathetic activity in the IDH and non-IDH groups.

Parameters	IDH	Non-IDH	*P*
**Plasma renin activity (ng**∙**mL**^**-1**^∙**h**^**-1**^**)**			
Pre-dialysis	2.40 ± 2.56	3.86 ± 4.85	0.130
Post-dialysis	3.23 ± 4.89	6.26 ± 7.30	0.730
**Serum aldosterone (nmol/L)**			
Pre-dialysis	3.1 ± 6.5	7.5 ± 13.6	0.009
Post-dialysis	64.4 ± 79.1	261.7 ± 478.4	0.005
**Serum epinephrine (pmol/L)**			
Pre-dialysis	247.6 ± 120.8	221.8 ± 114.4	0.478
Post-dialysis	175.6 ± 61.6	197.9 ± 105.0	0.310
**Serum norepinephrine (pmol/L)**			
Pre-dialysis	1518.1 ± 1231.8	1456.6 ± 1135.4	0.867
Post-dialysis	921.4 ± 995.9	843.3 ± 835.6	0.789

Data are presented as mean ± standard deviation.

IDH, intra-dialytic hypertension.

### Mortality

**Fig 2:**
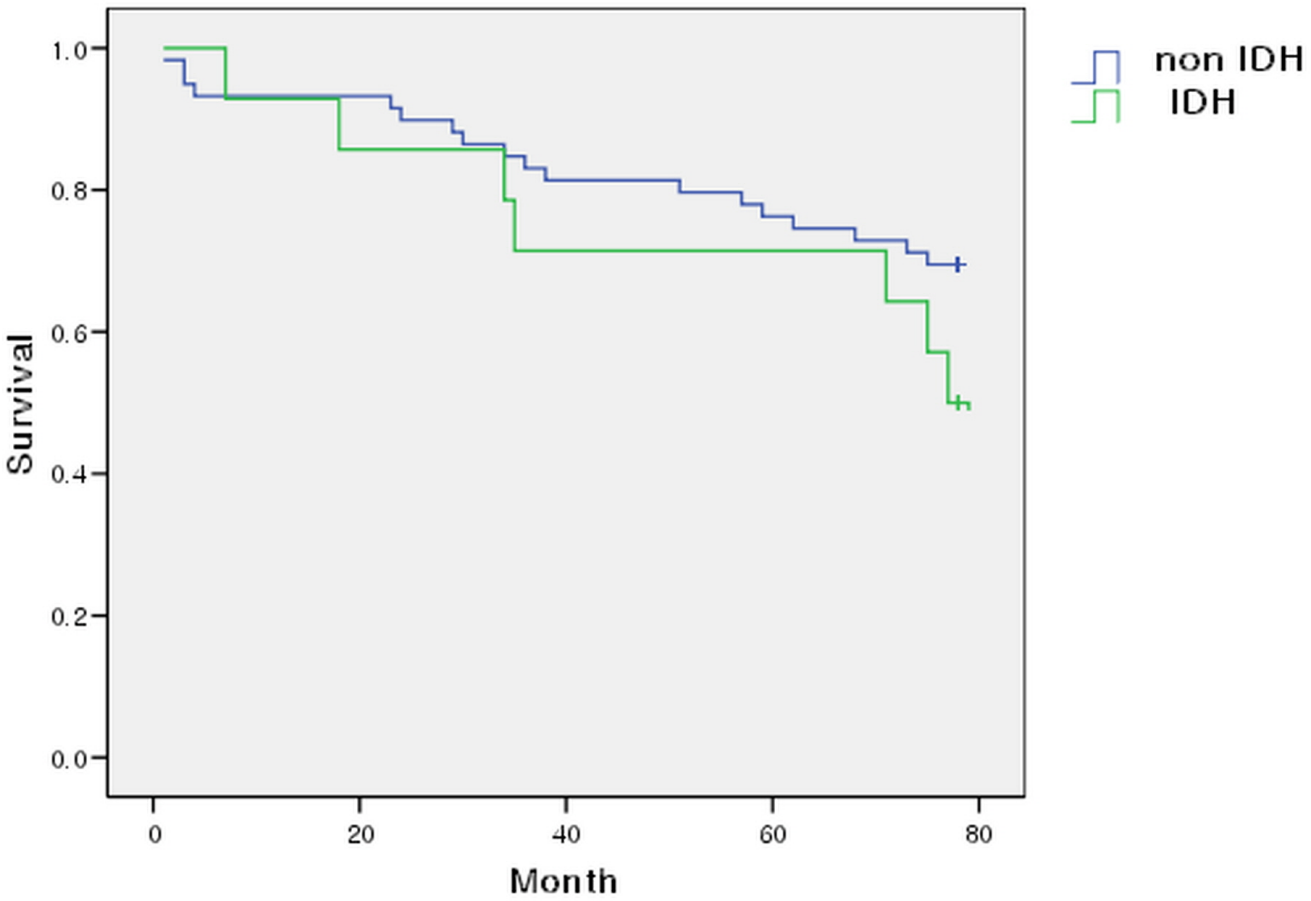
Kaplan Meier overall survival curve. The non-IDH group had higher survival rate than the IDH group. However, there was no significant difference in overall survival rate between the IDH group and the non-IHD group (50% vs. 69.5%, *P* = 0.209).

During 78 months of follow-up, 26 patients died, including 8 patients in the IDH group (6 males and 2 females) and 18 patients in the non-IDH group (10 males and 8 females). The overall survival in Kaplan-Meier plots for the non-IDH group was higher than that in the IDH group. However, the overall survival rates in the two groups were not significantly different (50% vs 69.5%, *P* = 0.209) (Fig 2). Univariate Cox regression was done for factors with P < 0.05 or those that were clinically significant (Table 5). The results of multivariate Cox regression analysis showed that the IDH Group had 2.846 times higher mortality rate than non IDH Group (Table 6). The reason Kaplan-Meier plots and multivariate Cox regression differed was the compounding effect. Hypertensive patients were divided into four groups. In Group 1, BP was within the normal range and no antihypertensive drugs were prescribed. In Group 2, control of BP required antihypertensive drugs from only one category. In Group 3, two or three categories of antihypertensive drug were required to control BP, but not minoxidil. In Group 4, antihypertensive drugs from more than three categories were required to control BP, including minoxidil.

**Table 5:** Univariate Cox regression analysis for mortality.

Parameters	P
Weight. change	0.24
Age	0.00036
IDH	0.21
HTN grade	0.11
Grade 1	0.056
Grade 2	0.70
Grade 3	0.89
DM	0.65
Hemoglobin	0.025
Erythropoietin dose	0.27
HCO3	0.059
nPCR	0.33
BUN	0.64
Cr	0.08

nPCR, normalized protein catabolic rate; IDH, intra-dialytic hypertension; BUN, blood urea nitrogen; Cr, creatinine.

**Table 6:** Multivariate Cox regression analysis for mortality (Hazards ratio, 95% confidence intervals).

Parameters	P	Hazard Ratios(95% confidence intervals)
Age	0.001	1.095(1.040–1.153)
HTN grade	0.004	
Grade 1	0.002	12.439(2.560–60.439)
Grade 2	0.127	3.049(0.729–12.744)
Grade 3	0.035	9.475(1.166–76.991)
Albumin	-	-
HCO3	0.09	1.127(0.979–1.298)
IDH	0.034	2.846(1.081–7.490)
Creatinine	-	-
DM	-	-

HTN, hypertension; IDH, intra-dialytic hypertension; DM, diabetes mellitus

## Discussion

This study has limitations in its sample size. However, it demonstrated that low serum potassium levels, low ultrafiltration, and low AMA in patients are risk factors for IDH. To the best of our knowledge, this is the first report that demonstrates that serum potassium level, ultrafiltration volume, and AMA are associated with IDH in hemodialysis patients.

As serum potassium is a uremic retention solute, clinicians aim to maintain low values of the solute. However, our findings imply that very low values of serum potassium level might be harmful. Therefore, a safe cut off level is required to ensure patients are not over-treated.

Although the relationship between potassium level and hypertension has not been clearly defined, several hypotheses have been proposed in previous studies, including low potassium intake, serum potassium in natriuresis, antioxidant action, correction of insulin resistance, and inhibition of sympathetic activity [18–22]. However, the pathogenetic role of low potassium level in IDH was not determined in the present study. Further studies are required to confirm the relationship between potassium level and IDH.

Nutrition is another important issue among hemodialysis patients. AMA and nPCR are nutritional indices correlated with the prognosis of hemodialysis patients [23,24]. The Kidney Disease Outcomes Quality Initiative guidelines recommend the measurement of serum urea nitrogen level for evaluating the nutritional status in ESRD patients. Presently, serum urea nitrogen, BMI, AMA, and nPCR values were significantly lower in the IDH group. Because urea nitrogen and AMA values are positively correlated with protein intake, low AMA and low urea nitrogen values in IDH patients would suggest low protein intake. Similarly, the lower nPCR values in IDH patients are indicative of low protein intake because nPCR is dependent on protein intake and Kt/V urea. In this study, Kt/V urea differed significantly between the two groups (0.69 ± 0.12 *vs*. 0.86 ± 0.24 g∙kg^-1^∙day^-1^, *P* = 0.011). These lower values of serum urea nitrogen, BMI, AMA, and nPCR in IDH patients suggest that IDH patients have poorer nutritional status.

As seen in Table 2, the ultrafiltration volume in the IDH group was lower than that in the non-IDH group. However, the ratio of ultrafiltration volume to inter-dialytic weight gain did not differ significantly between the two groups (1.20 ± 0.34 *vs*. 1.18 ± 0.21 L/kg, *P* = 0.86). Therefore, a lower ultrafiltration volume cannot explain the development of IDH.

The most popular hypotheses to explain IDH are stimulation of renin-angiotensin system and the overactivation of sympathetic system by ultrafiltration induced hypovolemia. In this study, the pre- and post- dialytic aldosterone levels were lower in the IDH group than the non-IDH group. No significant differences of epinephrine and norepinephrine level were present (Table 3). In IDH patients, post-dialytic SBP is higher than pre-dialytic SBP. Therefore, intra-dialytic aldosterone secretion is attenuated by elevated blood pressure during hemodialysis. Considering consecutive sessions for the maintenance hemodialysis, serum aldosterone level in IDH patients could be lower than that in non-IDH patients before and after dialysis. Although epinephrine and norepinephrine level were not significantly different, another method to explore the sympathetic nervous system overactivation is needed.

Peripheral vascular resistance is increased in IDH patients [25]. Peripheral resistance increases in IDH patients are associated with increased endothelin [25, 26]. The mechanism of endothelin increase in IDH patients is not clear. Unfortunately, this study did not measure endothelin levels. Studies of vasoconstriction peptides, including entothelin, may help understand IDH mechanism and manage IDH patient.

Increased blood viscosity increase peripheral resistance and cause hypertension. In this study, haematocrit and erythropoietin dose were not associated with IDH. But, progressive ultrafiltration induced rise of hematocrit because of the hemoconcentration can cause IDH. So there is a need to study the relationship between pre-dialytic and post-dialytic hematocrit changes and IHD. Presently there was no association between erythropoietin dose and IDH, However, care is advised before prescribing erythropoietin because erythropoietin triggers endothelin synthesis [27].

IDH is associated with poor outcomes [8,18]. In the present study, the overall mortality in the IDH group was higher than that in the non-IDH group, although there was no significant difference in cumulative mortality between the two groups (Fig 2). The small number of study subjects and relatively short period of follow-up might have caused the lack of significance. Additional studies with larger size and longer follow-up period are required.

The present study is retrospective in nature. It involved a review of medical records. The limitations of this study include its small sample size, single-centre nature, and observational design. Further well-designed prospective studies are needed to confirm our findings.
